# Surgical Treatment for Stress Urinary Incontinence in Women: A Systematic Review and Meta-analysis

**DOI:** 10.1055/s-0038-1667184

**Published:** 2018-08

**Authors:** Letícia Maria de Oliveira, Marcia Maria Dias, Sérgio Brasileiro Martins, Jorge Milhem Haddad, Manoel João Batista Castello Girão, Rodrigo de Aquino Castro

**Affiliations:** 1 Department of Gynecology, Paulista School of Medicine, Universidade Federal de São Paulo, São Paulo, SP, Brazil; 2 Department of Gynecology, Faculty of Medicine, Universidade de São Paulo, São Paulo, SP, Brazil

**Keywords:** stress urinary incontinence, Burch surgery, midurethral sling, pubovaginal sling, meta-analysis, incontinência urinária de esforço, cirurgia de Burch, *sling* de uretra média, *sling* pubovaginal, metanálise

## Abstract

**Objective** To compare surgical treatments for stress urinary incontinence in terms of efficiency and complications.

**Data Sources** We searched the MEDLINE and COCHRANE databases using the terms *stress urinary incontinence*, *surgical treatment for stress urinary incontinence* and *sling*.

**Selection of Studies** Forty-eight studies were selected, which amounted to a total of 6,881 patients with scores equal to or higher than 3 in the Jadad scale.

**Data Collection** Each study was read by one of the authors, added to a standardized table and checked by a second author. We extracted data on intervention details, follow-up time, the results of treatment and adverse events.

**Data Synthesis** Comparing retropubic versus transobturator slings, the former was superior for both objective (odds ratio [OR], 1.27; 95% confidence interval [CI], 1.05–1.54) and subjective (OR, 1.23; 95% CI, 1.02–1.48) cures. Between minislings versus other slings, there was a difference favoring other slings for subjective cure (OR, 0.58; 95% CI, 0.39–0.86). Between pubovaginal sling versus Burch surgery, there was a difference for both objective (OR, 2.04; 95% CI, 1.50–2.77) and subjective (OR, 1.64; 95% CI, 1.10–2.44) cures, favoring pubovaginal sling. There was no difference in the groups: midurethral slings versus Burch, pubovaginal sling versus midurethral slings, transobturator slings, minislings versus other slings (objective cure). Retropubic and pubovaginal slings are more retentionist. Retropubic slings have more bladder perforation, and transobturator slings, more leg and groin pain, neurological lesion and vaginal perforation.

**Conclusion** Pubovaginal slings are superior to Burch colposuspension surgery but exhibit more retention. Retropubic slings are superior to transobturator slings, with more adverse events. Other slings are superior to minislings in the subjective aspect. There was no difference in the comparisons between midurethral slings versus Burch colposuspension surgery, pubovaginal versus midurethral slings, and inside-out versus outside-in transobturator slings.

## Abstract (pt)

**Resumo:**

**Objetivo** comparar tratamentos cirúrgicos para incontinência urinária de esforço (IUE), quanto à eficiência e complicações, por meio de revisão sistemática seguida de metanálise.

**Fonte dos dados** Fizemos busca nas bases de dados MEDLINE e COCHRANE, utilizando os termos *stress urinary incontinence*, *surgical treatment for stress urinary incontinence* e *sling*.

**Seleção dos estudos** Selecionamos 48 estudos, totalizando 6.881 pacientes com pontuação igual ou maior do que 3 na escala de Jadad.

**Coleta de dados** Cada estudo foi lido por um autor, colocado em tabela, e checado por outro autor. Extraímos dados como detalhes das intervenções, tempo de seguimento, resultados do tratamento e eventos adversos.

**Síntese dos dados** Não houve diferença nas comparações: *sling* de uretra média versus cirurgia de Burch, quanto às curas objetiva (razão de chances [RC]: 1,29; intervalo de confiança de 95% [IC95%]: 0,76–2,20) e subjetiva (RC: 1,16; IC95%: 0,67–2,00); *sling* de uretra média transobturatório *outside-in* versus *inside-out* quanto às curas objetiva (RC: 0,78; IC95%: 0,45–1,35) e subjetiva (RC: 0,83; IC95%: 0,58–1,18); *sling* pubovaginal e de uretra média quanto à cura objetiva (RC: 1,64; IC 95%: 0,52–5,15). Comparando *sling* retropúbico com transobturatório, o retropúbico foi superior quanto às curas objetiva (RC: 1,27; IC95%: 1,05–1,54) e subjetiva (RC: 1,23; IC95%: 1,02–1,48). Entre *minislings* e outros *slings*, houve diferença favorável a outros *slings* quanto à cura subjetiva (RC: 0,58; IC95%: 0,39–0,86) mas não quanto à cura objetiva (RC: 0,72; IC95%: 0,47–1,10). No grupo *sling* pubovaginal e Burch, houve diferença quanto à cura objetiva (RC: 2,04; IC95%: 1,50–2,77) e subjetiva (RC: 1,64; IC95%: 1,10–2,44). *Slings* de uretra média apresentam mais erosão, enquanto a cirurgia de Burch tem mais complicações na ferida operatória e infecção do trato urinário. *Slings* retropúbicos e pubovaginais são mais retencionistas. *Slings* retropúbicos estão mais associados a lesão vascular, hematomas e perfuração vesical, e transobturatórios, à dor na perna e virilha, lesão neurológica e perfuração vaginal.

**Conclusão** *Slings* pubovaginais são superiores à cirurgia de Burch, porém mais retencionistas. *Slings* retropúbicos são superiores aos transobturatórios, embora tenham mais eventos adversos. Outros *slings* são superiores aos minislings em relação ao aspecto subjetivo. Não houve diferença nas comparações entre *slings* de uretra média e cirurgia de Burch, *slings* pubovaginais, transobturatórios *inside-out* e *inside-in*.

## Introduction

Stress urinary incontinence (SUI) is defined by the International Continence Society (ICS) as the involuntary loss of urine during physical exertion, such as while coughing, sneezing, laughing or running.^1^ This condition affects 13 to 46% of women at a young age, reaching even higher rates if we consider postmenopausal women,^2^ ^3^ ^4^ with severe repercussions for quality of life as it affects physical, sexual, emotional and social aspects.^3^

Several clinical and surgical options have been used for the treatment of SUI. Our review does not take into account clinical treatments. For surgical treatments, several techniques are described, including the more commonly known: Burch colposuspension, either abdominal or laparoscopic, pubovaginal slings, retropubic and transobturator midurethral slings, and single-incision slings (minislings).

Burch colposuspension and pubovaginal slings are considered the “gold standard” for surgical treatment of SUI. Since described in 1996 by Ulmsten et al,^5^ the synthetic tension-free vaginal tape (TVT) sling has been used in a growing and widespread manner throughout the world. Even though this technique has achieved high cure rates in the mid and long term,^6^ ^7^ important complications, such as bladder perforation, retropubic hematomas and voiding dysfunction have also been reported.^8^ ^9^ In an attempt to minimize these complications, in 2001, Delorme^10^ described a new technique involving the placement of a synthetic mesh under the middle urethra through the transobturator route from the thigh to the vagina (transobturator tape outside-in [TOT]). In 2003, de Leval^11^ introduced a modification to the technique, proposing insertion of the mesh toward the opposite direction, from the vagina to the thigh (transobturator tape inside-out [TVT-O]). Both slings placed by transobturator approach have shown high rates of cure.^10^ ^11^ However, several researchers describe thigh pain as a main complication.^12^ Thus, to further reduce complication rates, single-incision slings, or minislings, were introduced with objective and subjective cure rates very close to those obtained with TVT and TOT at mid-term follow-up, according to a meta-analysis published in 2014.^13^

The literature is vast regarding surgical procedure success rates for treating female SUI, but the quality of many studies is questionable. In an attempt to clarify the best technique for each case, we proposed this systematic review followed by meta-analysis, based on good quality randomized trials, comparing objective and subjective results, and complications.

## Study Search

We searched the MEDLINE and Cochrane Central Register for Controlled Trials databases from January 1990 to December 2016. We used the following keywords to search for studies: *stress urinary incontinence*, *surgical treatment for stress urinary incontinence*, *sling*, *pubovaginal sling*, *retropubic sling*, *transobturator sling*, *minisling*, *Burch colposuspension*. The search was limited to comparative and randomized studies. We included only human studies written in English, French and Spanish. We found some few articles in other languages that did not fill the Jadad criteria. The articles listed in the search results were only used when the full text was available. The authors of the studies were not contacted.

Three of the authors (LMO, MMD, SBM) in our meta-analysis did the initial research of all studies independently. After reading the titles and abstracts, we read the full text of the studies considered potentially eligible, which were later included in a standardized table for data extraction if the eligibility criteria were met.

## Study Selection

We selected the relevant studies by applying the three-point questionnaire that form the basis he Jadad scale. Each question was to be answered with either a *yes* or a *no*. Each *yes* would score a single point, each *no* zero points The questions were as follows: *Was the study described as randomized?*; *Was the study described as double blind?* and *Was there a description of withdrawals and dropouts?* To receive the corresponding point, an article should describe the number of withdrawals and dropouts, in each of the study groups, and the underlying reasons. Additional points were given if: *The method of randomization was described in the paper, and that method was appropriate* or *The method of blinding was described, and it was appropriate.* Points would be deducted if: *The method of randomization was described but was inappropriate.* or *The method of blinding was described, but was inappropriate.* A clinical trial could therefore receive a Jadad score between zero and five.^14^ Studies with a score lower than three points on this scale were excluded.

To evaluate the results, we included randomized, comparative studies with a minimum of 12 months of follow-up, comparing 2 or more sling procedures or a sling procedure with Burch colposuspension surgery, performed on women over 18 years of age with SUI diagnosed by clinical history, stress test and/or urodynamic evaluation or *pad test.* Studies that included mixed urinary incontinence (MUI), predominantly SUI, and intrinsic sphincteric deficiency (ISD) were also admitted.

Whenever there were three arms in the study, we compared two arms at a time. For the analysis of side effects, we used only the studies that were selected for the meta-analysis.

The types of slings included were midurethral slings (retropubic and transobturator), pubovaginal slings (synthetic and autologous) and minislings.

Studies comparing the Burch technique with any other non-sling surgical modality to treat SUI were not included.

Studies using materials that were withdrawn from the market were excluded from our review, as were studies comparing different products by equal routes.

The results of interest in the studies analyzed were divided into six categories: objective or subjective cure, perioperative results, quality of life and satisfaction questionnaires, sexual function and adverse events (Table 1). However, only meta-analytic studies were performed for objective or subjective cure and adverse events.

**Table 1:** Randomized controlled trials included in the systematic review

Study	Intervention (1)	Comparator (2)	N (1)	N (2)	Follow-up	OC	SC	PO	AE	QoL	SF
MUS versus Burch											
Bai et al. (2005)^15^	Retropubic (TVT)	Burch	31	33	1 year	X			X		
Jelovsek et al. (2008)^16^	Retropubic (TVT)	Burch L	25	28	65 months		X			X	
Liapis et al. (2002)^17^	Retropubic (TVT)	Burch	35	36	2 years	X		X	X		
Paraiso et al. (2004)^18^	Retropubic (TVT)	Burch lap	31	32	21 months	X		X	X	X	
Persson et al. (2002)^19^	Retropubic (TVT)	Burch lap	37	31	1 year	X	X	X	X		
Ward et al. (2008)^20^	Retropubic (TVT)	Burch	72	49	5 years	X		X	X	X	X
Valpas et al. (2015)^21^	Retropubic (TVT)	Burch lap	51	40	5 years	X	X	X	X	X	
**PVS versus Burch**											
Albo et al. (2007)^22^	PVS (autologous fascia)	Burch	326	329	2 years	X	X	X	X	X	
Bai et al. (2005)^15^	PVS (autologous fascia)	Burch	28	33	1 year	X			X		
Culligan et al. (2003)^23^	PVS (Gore-Tex)	Burch	13	15	73 months	X	X	X	X		
**PVS versus MUS**											
Bai et al. (2005)^15^	PVS (autologous fascia)	Retropubic (TVT)	28	31	1 year	X			X		
Guerrero et al. (2010)^24^	PVS (autologous fascia)	Retropubic (TVT)	67	69	1 year		X	X	X	X	
Sharifiaghdas and Mortazavi (2008)^25^	PVS (autologous fascia)	Retropubic (TVT)	25	36	40 months	X	X	X	X	X	
**TVT versus TOT**											
Angioli et al. (2010)^26^	TVT	TVT-O	35	37	5 years	X	X	X	X	X	X
Araco et al. (2008)^27^	TVT	TVT-O	108	109	1 year	X		X	X	X	
Barber et al. (2008)^28^	TVT	Monarc	79	71	1 year	X	X	X	X	X	X
Costantini et al. (2016)^29^	TVT	Obtape	40	47	5 years	X	X	X	X	X	
Deffieux et al. (2010)^30^	TVT	TVT-O	67	65	2 years	X	X	X	X	X	X
Freeman et al. (2011)^31^	TVT	Monarc	85	95	1 year		X	X	X	X	X
Karateke et al. (2009)^32^	TVT	TVT-O	81	83	14 months	X	X	X	X		
Krofta et al. (2010)^33^	TVT	TVT-O	141	147	1 year	X	X	X	X	X	X
Laurikainen et al. (2014)^34^	TVT	TVT-O	131	123	5 years	X	X	X	X		
Lee et al. (2007)^35^	TVT	TVT-O	60	60	13 months	X	X	X	X	X	
Richter et al. (2010)^36^	TVT	TVT-O/Monarc	291	292	1 year	X	X	X	X	X	X
Rinne et al. (2008)^37^	TVT	TVT-O	134	131	1 year		X	X	X	X	
Ross et al. (2009)^38^	Advantage	Obtrix	95	86	1 year	X	X	X	X	X	X
Ross et al. (2016)^39^	Advantage	Obtrix	74	66	5 years	X			X	X	X
Scheiner et al. (2012)^40^	TVT	Monarc	65	34	1 year	X	X	X	X	X	X
Scheiner et al. (2012)^40^	TVT	TVT-O	65	37	1 year	X	X	X	X	X	X
Schierlitz et al. (2012)^41^	TVT	TVT-O	72	75	3 years	X	X	X	X	X	
Teo et al. (2011)^42^	TVT	TVT-O	41	29	1 year	X	X	X	X	X	
Wadie and El-Hefnawy (2013)^43^	TVT	TOT (Aris)	36	35	2 years	X	X	X	X	X	
Wang et al. (2010)^44^	TVT	TOT	70	70	1 year	X	X	X	X	X	
Wang et al. (2009)^45^	TVT	TVT-O	35	30	3 years	X		X	X		
Zhang et al. (2016)^46^	TVT	TVT-O	58	62	95 months	X	X	X	X	X	
**TOT versus TVT-O**											
Abdel-Fattah et al. (2010)^47^	TOT (Aris)	TVT-O	152	147	1 year	X	X		X	X	X
Houvert et al. (2009)^48^	TOT (Monarco)	TVT-O	86	75	38 months		X	X	X	X	X
Liapis et al. (2008)^49^	TOT (Monarc)	TVT-O	53	61	1 year	X	X	X	X		
Park and Kim (2012)^50^	Monarc	TVT-O	35	39	3 years	X	X	X	X		
Scheiner et al. (2012)^40^	Monarc	TVT-O	34	37	1 year	X	X	X	X	X	X
**Minisling versus any sling**											
Basu and Duckett (2013)^51^	Miniarc	Retropubic (Advantage)	38	33	3 years		X	X	X	X	
Djehdian et al. (2014)^52^	Ophira	TOT (Unitape)	69	61	1 year	X	X	X	X	X	
Gaber et al. (2016)^53^	Contasure-Needleless	TVT-O	70	70	1 year	X	X	X	X	X	
Gaber et al. (2016)^53^	EFA	TVT-O	69	70	1 year	X	X	X	X	X	
Jurakova et al. (2016)^54^	Ophira	TVT-O	44	46	1 year	X	X	X	X	X	
Lee et al. (2015)^55^	Miniarc	TOT (Monarc)	103	103	1 year	X	X	X	X		
Schellart et al. (2016)^56^	Miniarc	TOT (Monarc)	73	72	2 years	X	X	X	X	X	
Sivaslioglu et al. (2012)^57^	TFS	TOT (I-STOP)	36	36	5 years	X	X	X	X		

Abbreviations: EFA, endopelvic free anchor; MUS, midurethral sling; PVS, pubovaginal sling; TFS, tissue fixation system; TOT, transobturator tape; TVT, tension-free vaginal tape; TVT-O, tension-free vaginal tape obturator.

## Data Extraction and Assessment

Each of the included studies was read by one of the authors, and the data were extracted and inserted in a previously standardized table. Then, each study was checked by a second author. Discrepancies were resolved by consensus among three of the authors. We extracted data on study characteristics, details of interventions, follow-up time, results of treatment and adverse events.

## Data Synthesis and Analysis

We compared midurethral sling versus Burch surgery, pubovaginal sling versus Burch surgery, pubovaginal sling versus midurethral sling, retropubic versus transobturator midurethral sling, transobturator outside-in midurethral sling versus transobturator inside-out and minisling versus other slings.

Whenever we found two or more randomized studies comparing the same surgical techniques in relation to the same outcomes and adverse events, we resorted to a meta-analysis, which is the most adequate statistical technique to combine results from different studies.^58^ ^59^

It is natural to think of using the fixed-effect model, which assumes that the effect of interest is the same in all included studies. However, the studies are not identical regarding effect of interest and are therefore considered heterogeneous. Thus, to verify the existence of heterogeneity, we used the Cochran Q test and the I^2^ statistic by Higgins and Thompson.^60^

The null hypothesis of the Cochran Q test asserts that the studies are homogeneous. A high Q value indicates that there is great heterogeneity. However, the *p*-value associated with the test indicates whether the heterogeneity is significant or not, if different from zero. A deficiency of this test is its low power when the meta-analysis is made up of a small number of studies. The I^2^ statistic by Higgins and Thompson^60^ derives from Cochran Q test and the number of studies involved in the meta-analysis. The I^2^ statistic can range from minus zero to 100%. Negative values are considered zero. The *p*-value of I^2^ is equivalent to the *p*-value of Cochran Q test.^60^

Higgins and Thompson^60^ suggest a scale where a value of I^2^ close to zero indicates that there is no heterogeneity between studies, while a value close to 25% indicates low heterogeneity, 50% indicates moderate heterogeneity, and more than 75% indicates high heterogeneity.^60^

Just as in the option of effect measure, we used odds ratio (OR). We used the Mantel-Haenszel method because most of the studies included had small sample sizes. However, for certain effects, some studies presented zero events in at least one of the comparison groups, and in these cases, we used the Peto method.^61^

We used the Cochrane Collaboration's Review Manager software (RevMan, The Nordic Cochrane Centre, The Cochrane Collaboration, Copenhagen, Denmark), version 5.3, to conduct our meta-analysis.

## Results

**Fig. 1:**
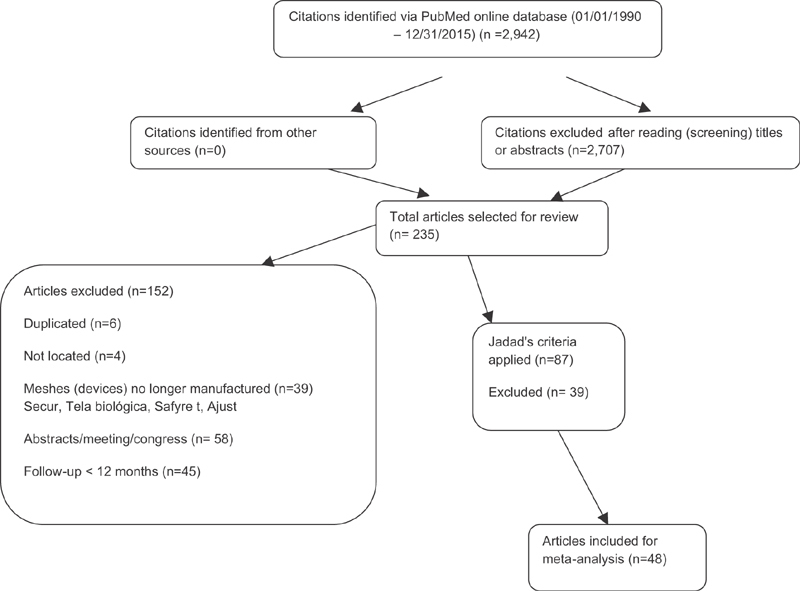
Flowchart.

The searches performed on MEDLINE and Cochrane resulted in 2,942 abstracts. After reading the titles and abstracts, 2,707 results were excluded and there were 235 remaining, whose texts were read in full. The study search flow is detailed in Fig. 1. Next, we found 48 articles that met the inclusion criteria in the meta-analysis, totaling 6,881 patients, Table 1.

### Midurethral Sling versus Burch

For this comparison, we found 7 studies that analyzed 531 patients (282 in the midurethral sling group and 249 in the Burch surgery group). All studies used the Gynecare TVT retropubic sling (Ethicon Inc., Somerville, New Jersey, USA), compared with laparotomy^17^ or laparoscopic^16^ ^21^ Burch surgery. Of the studies included in this group, six yielded objective cure results,^15^ ^17^ ^18^ ^19^ ^20^ ^21^ while three presented data on subjective cure,^16^ ^19^ ^21^ and six presented data on adverse events, with the exception of Jelovsek et al (2008).^16^

The following tests were used to assess objective cure: pad test,^17^ ^19^ ^20^ stress test^15^ ^21^ and urodynamic evaluation.^17^ ^18^ ^19^ ^20^

For subjective cure, the authors used: satisfaction questionnaire,^19^ visual analog scale(VAS),^18^ ^21^ Urinary Incontinence Severity Score (UISS),^21^ Patients Global Impression of Improvement (PGII),^16^ ^21^ Incontinence Severity Index (ISI),^16^ Urogenital Distress Inventory 6 (UDI-6),^16^ ^18^ Incontinence Impact Questionnaire 7 (IIQ-7),^16^ ^18^ Bristol Female Lower Urinary Tract Symptoms (BFLUTS)^ 20^ and Short Form-36 (SF-36).^20^

**Fig. 2:**
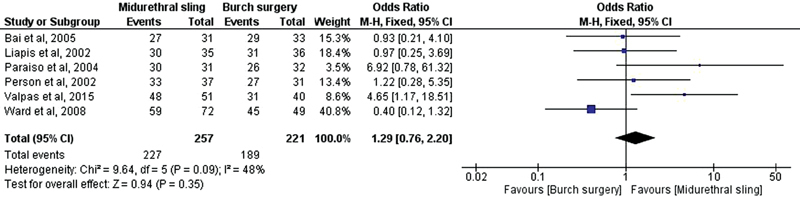
Objective cure: midurethral slings versus Burch surgery.

**Fig. 3:**
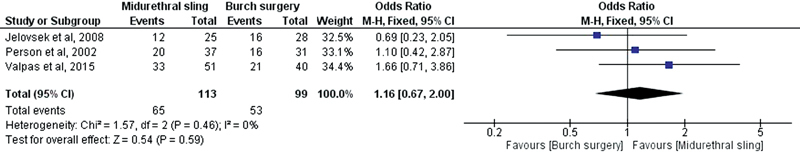
Subjective cure: midurethral slings versus Burch surgery.

The meta-analysis showed no significant difference regarding objective cure in the comparison between midurethral sling and Burch surgery (OR, 1.29; 95% confidence interval [CI], 0.76–2.20) Fig. 2. Moreover, no significant difference was found for subjective cure (OR, 1.16; 95% CI, 0.67–2.00) Fig. 3.

Regarding adverse events, we observed that the midurethral slings had higher rates of erosion (OR, 5.98; 95% CI, 1.16–30.67) and bladder perforation (OR, 2.74; 95% CI, 1.24–6.03), while Burch surgery had higher rates of surgical wound complications (OR, 0.30; 95% CI, 0.10–0.90) and urinary tract infection (UTI) (OR, 0.30; 95% CI, 0.14–0.63). There was no significant difference between these procedures in relation to the following adverse events: postoperative pain, hematoma, need for further surgery due to erosion or urinary retention, urinary retention for less than 6 weeks and overactive bladder. Adverse events such as blood loss, retention lasting for longer than 6 weeks, transfusion, de novo urgency and vaginal perforation were described in a single study, and therefore did not justify a meta-analysis.

### Pubovaginal Sling versus Burch Surgery

For this comparison, we found 3 studies with high-quality evidence including 744 patients; 367 in the pubovaginal sling group and 377 in the Burch group. Two studies used autologous rectus fascia^15^ ^22^ and one study used a synthetic sling.^23^ The three were compared with laparotomy Burch colposuspension. All the studies in this group presented results for objective cure and adverse events, while only two showed data on subjective cure.^22^ ^23^ To assess objective cure, the following tests were used: pad test^22^ ^23^ and stress test.^15^ ^22^ ^23^ To assess subjective cure, the authors used: UDI and IIQ.^22^

**Fig. 4:**
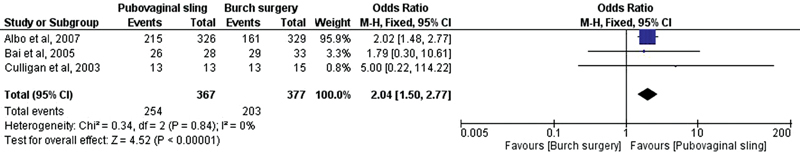
Objective cure: Pubovaginal sling versus Burch surgery.

The meta-analysis showed no statistically significant difference regarding objective cure in the comparison between pubovaginal slings and Burch colposuspension (OR, 2.04; 95% CI, 1.50–2.77) (Fig. 4).

**Fig. 5:**
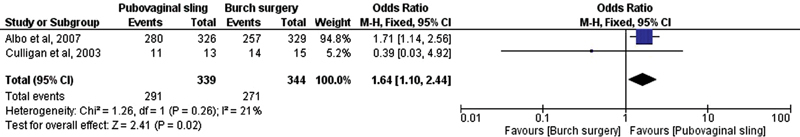
Subjective cure: Pubovaginal sling versus Burch surgery.

Regarding subjective cure, the meta-analysis results showed a significant difference favoring pubovaginal slings over Burch colposuspension (OR, 1.64; 95% CI, 1.10–2.44) (Fig. 5).

Regarding adverse events, we observed that, according to the analysis, the patients returned to the operating room more often due to retention in the group of pubovaginal slings, showing statistical significance (OR, 7.95; 95% CI, 3.34–18.94). Other complications were included in a single study, which precludes a meta-analytical comparison.

### Pubovaginal Sling versus Midurethral Sling

**Fig. 6:**
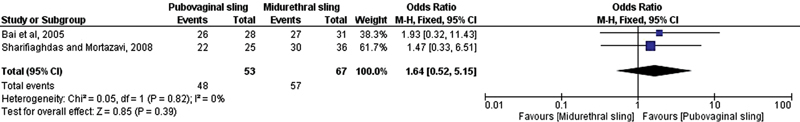
Objective cure: Pubovaginal sling versus midurethral sling.

For this comparison, we selected 3 studies including 256 patients, 120 in the pubovaginal sling group and 136 in the midurethral sling group. In all studies, autologous rectus fascia was used to construct a pubovaginal sling. For midurethral sling, all studies used retropubic TVT. Of the studies found in this group of analysis, two presented results on objective cure^15^ and only one showed subjective cure.^24^ To assess objective cure, the authors used: the pad test,^25^ stress test^15^ ^25^ and urodynamic evaluation.^25^ To analyze subjective cure, the authors used: the satisfaction test,^24^ BFLUTS^24^ and IIQ.^25^ Regarding objective cure, the meta-analysis showed that there was no significant between-group difference (OR 1.64, 95% CI: 0.52–5.15) (Fig. 6). For subjective cure, there was no possibility of meta-analysis, since this variable was analyzed in one study only.

In this group, we observed that some adverse events were reported, such as bladder perforation, urinary retention for less than 6 weeks and return to the operating room due to urinary retention, although these were not significant between groups. Other complications, such as blood loss, transfusion, and de novo urgency were described in a single study, and therefore did not justify a meta-analysis.

### Retropubic Sling versus Transobturator Sling

In this comparison group, we found 22 studies including 3,638 patients, 1,863 in the group treated with retropubic sling and 1,775 in the transobturator group. In most of them, the Gynecare TVT and TVT-O (Ethicon Inc., Somerville, New Jersey, USA) were compared.^26^ ^27^ ^30^ ^32^ ^33^ ^34^ ^35^ ^37^ ^40^ ^41^ ^42^ ^46^ ^47^ In the other studies, TVT and Monarc (American Medical Systems, Minnetonka, MN, USA) were compared.^28^ ^31^ ^40^ ^44^ One study, by Richter et al (2010),^36^ compared TVT with TVT-O or Monarc. Ross et al (2009, 2016)^38^ ^39^ used Advantage (retropubic) and Obtrix (transobturator) (both products made by Boston Scientific, Natick, MA, USA), and Wadie and El-Hefnawy (2013)^43^ compared TVT and Aris TOT (Coloplast, Minneapolis, MN, USA). Tension-free vaginal tape and Obtape (Mentor-Porgés, Le Plessis-Robinson, France) were compared by Costantini et al (2016).^29^

Of the studies found in this comparison group, only one^31^ did not present results for objective cure. Six studies did not assess subjective cure.^27^ ^32^ ^37^ ^39^ ^41^ ^46^ All authors reported data on complications.

To assess objective cure, the authors used: the pad test,^29^ ^33^ ^34^ ^36^ ^37^ ^38^ ^39^ ^40^ ^42^ ^43^ ^45^ ^47^ stress test^26^ ^27^ ^28^ ^29^ ^30^ ^32^ ^33^ ^34^ ^35^ ^36^ ^37^ ^40^ ^42^ ^43^ ^44^ ^45^ and urodynamic evaluation.^26^ ^27^ ^32^ ^41^

To evaluate subjective cure, the authors used the following tools: satisfaction test,^30^ ^32^ ^33^ ^36^ ^37^ ^38^ VAS,^26^ ^30^ ^33^ ^34^ ^37^ ^40^ and quality of life questionnaires, including the Incontinence Quality of Life questionnaire (I-QOL),^27^ ^35^ ISI,^28^ Pelvic Floor Distress Inventory, Short Form-20 (PFDI-20),^28^ Pelvic Floor Impact Questionnaire Short Form-7 (PFIQ-7),^28^ ^47^ PGII,^28^ ^43^ ^47^ Short Form 12 (SF-12),^28^ Pelvic Organ Prolapse/Urinary Incontinence Sexual Questionnaire Short Form (PISQ-12),^28^ ^39^ ^47^ Quality Of Life Assessment Questionnaire Concerning Urinary Incontinence (CONTILIFE),^30^ ^33^ International Consultation Incontinence Modular Questionnaire-Female Lower Urinary Tract Symptoms (ICIQ-FLUTS),^31^ IIQ-7,^29^ ^32^ ^34^ ^37^ ^38^ ^39^ ^41^ ^42^ ^43^ UDI-6,^29^ ^32^ ^34^ ^37^ ^38^ ^39^ ^41^ ^42^ ^43^ UISS,^34^ ^37^ Detrusor Instability Score (DIS),^34^ ^37^ Medical Epidemiological and Social Aspects of Aging (MESA )36 and King's Health Questionnaire (KHQ).^40^

**Fig. 7:**
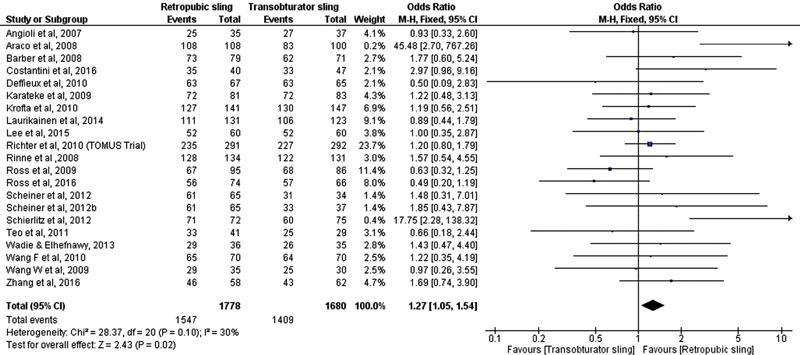
Objective cure: Retropubic sling versus transobturator sling.

**Fig. 8:**
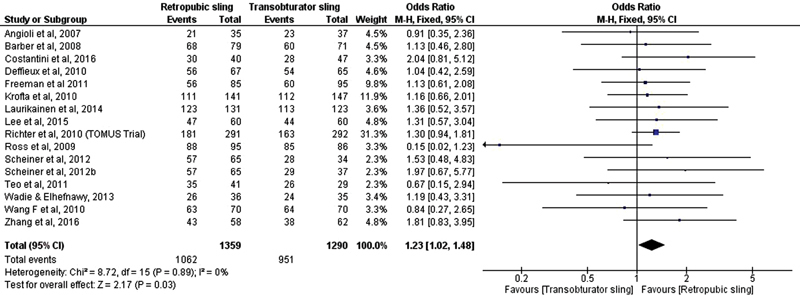
Subjective cure: Retropubic sling versus transobturator sling.

After the meta-analysis of objective cure data, the conclusion was that there was a statistically significant difference between the surgical treatments with retropubic and transobturator sling favoring the retropubic device (OR, 1.27; 95% CI, 1.05–1.54) Fig. 7. The same conclusions were drawn regarding subjective cure (OR, 1.23; 95% CI, 1.02–1.48) Fig. 8.

Regarding complications, the retropubic slings significantly caused a greater number of vascular lesions (OR, 2.96, 95% CI, 1.41–6.24), hematoma (OR, 3.02, 95% CI, 1.34–6.82), bladder perforation (OR, 5.45, 95% CI, 3.33–8.90), urinary retention for less than 6 weeks (OR, 2.00, 95% CI, 1.45–2.77) and return to the operating room due to urinary retention (OR, 3.78, 95% CI, 2.00–7.13). Surgical treatment of SUI using the transobturator sling, in turn, produced significantly more cases of all of the following: leg pain (OR, 0.18, 95% CI, 0.11–0.30), groin pain (OR, 0.17, 95% CI, 0.08–0.35), neurological injury (OR, 0.48, 95% CI, 0.27–0.87) and vaginal perforation (OR, 0.24, 95% CI, 0.14–0.40). There was no significant difference between these procedures related to the following adverse events: blood loss, overactive bladder, surgical wound complications, unspecified pain, erosion, return to the operating room due to erosion, urinary tract infection, blood transfusion, urethral perforation, urinary retention lasting for longer than 6 weeks and de novo urgency.

### Outside-in Midurethral Transobturator Sling versus Inside-out Midurethral Transobturator Sling

For this comparison, we found 5 studies totaling 719 patients, 360 in the TOT group and 359 in the TVT-O group. In one of the studies, the authors used an Aris TOT sling;^48^ TOT Monarc slings^40^ ^48^ ^49^ ^50^ were used in the other studies. These slings were compared with TVT-O slings.

Of the studies found in this group of analysis, four showed results on objective cure.^40^ ^47^ ^49^ ^50^ All of the studies presented data on subjective cure and adverse events.

To assess objective cure, the authors used: pad test,^40^ ^47^ ^49^ stress test^40^ ^50^ and urodynamic evaluation.^49^ ^50^

To assess subjective cure, the authors used: satisfaction test,^47^ VAS,^40^ and questionnaires on quality of life, including KHQ,^40^ ^47^ International Consultation on Incontinence Questionnaire-Short Form (ICIQ-SF),^47^ PGII,^47^ UDI-6^48^ and IIQ-7.^48^

**Fig. 9:**
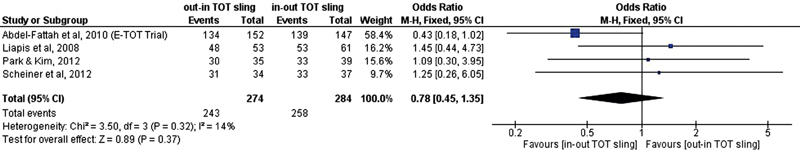
Objective cure: outside-in transobturator sling versus inside-out transobturator sling.

**Fig. 10:**
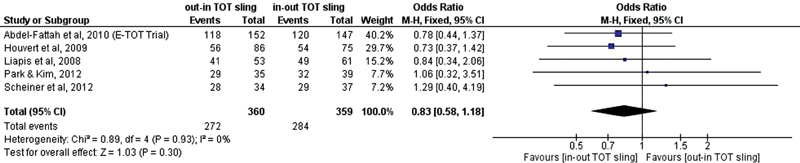
Subjective cure: outside-in transobturator sling versus inside-out transobturator sling.

The meta-analysis showed no significant difference regarding objective cure in the comparison between TOT and TVT-O slings (OR, 0.78, 95% CI, 0.45–1.35) Fig. 9. For subjective cure, no significant difference was found in the meta-analysis either (OR, 0.83; 95% CI, 0.58–1.18) Fig. 10.

Regarding adverse events, we observed that TOT slings presented higher rates of vaginal perforation (OR, 3.31, 95% CI, 1.44–7.61) and erosion (OR, 4.83, 95% CI, 1.28–18.27). There was no significant difference between these procedures in terms of postoperative pain, urinary retention for more or less than 6 weeks, return to the operating room due to urinary retention, de novo urgency and leg pain. Overactive bladder, UTI and urethral perforation were reported in a single study, thus precluding a meta-analysis.

### Minisling versus Any Other Sling

For this comparison, we found 8 studies totaling 993 patients, 502 in the minislings group and 491 in the comparison group. In three studies, the authors used the Miniarc minisling (American Medical Systems, Minnetonka, MN, USA),^51^ ^55^ ^56^ which was compared with the Advantage retropubic sling^51^ and the Monarc transobturator sling.^55^ ^56^ One of the studies compared the Ophira minisling and the TOT Unitape (both made by Promedon, Cordoba, Argentina),^52^ while others compared the Contasure-Needleless (New Medical Technologies, Barcelona, Spain) minisling and endopelvic free anchor (EFA),^53^ and the Ophira minisling^54^ with the TVT-O. One study compared the TFS minisling (TFS Surgical, Adelaide, Australia) with the TOT I-STOP (CL Medical, Sainte Foys Les Lyon, France).^57^

Only one study^51^ failed to report objective cure. All studies showed results for subjective cure and adverse events.

To assess objective cure, the authors used the pad test^52^ ^57^ and stress test.^52^ ^53^ ^54^ ^55^ ^56^

For subjective cure, the authors used the satisfaction test^52^ and quality of life questionnaires, including the KHQ,^51^ I-QOL,^52^ UDI,^52^ International Consultation on Incontinence Questionnaire/ Urinary Incontinence Short Form (ICIQ-UIFS),^53^ ^54^ ^55^ International Consultation on Incontinence Questionnaire/Overactive Bladder (ICIQ OAB),^55^ IIQ-7,^55^ PGII,^53^ ^54^ ^55^ ^56^ UDI-6,^56^ Patient Global Impression Severity (PGI-S),^56^ and Patient Perception of Intensity of Urgency Scale (PPIUS).^54^

**Fig. 11:**
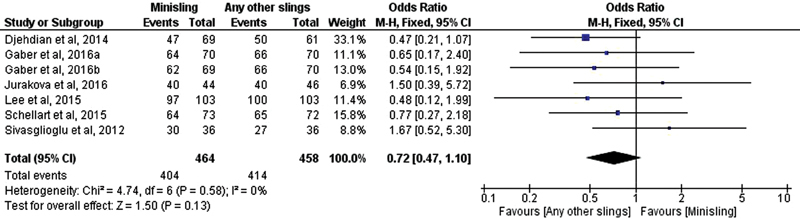
Objective cure: Minisling versus any other sling.

**Fig. 12:**
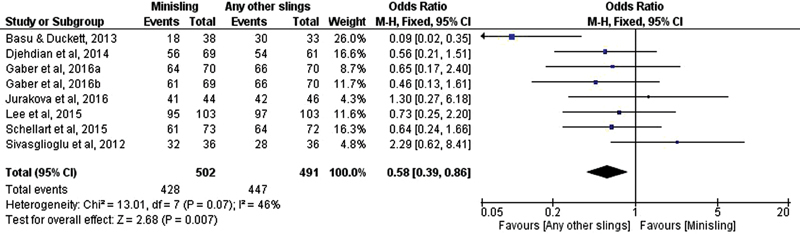
Subjective cure: Minisling versus any other sling.

The meta-analysis showed no significant difference between minislings and other slings for objective cure (OR, 0.72; 95% CI, 0.47–1.10) Fig. 11. For subjective cure, we found a significant difference favoring other slings (OR, 0.58, 95% CI, 0.39–0.86) Fig. 12.

Regarding the adverse events, the group that included other types of slings had a higher rate of groin pain (OR, 0.11 95% CI, 0.04–0.28) and unspecified pain (OR, 0.20, 95% CI, 0.07–0.61), noting that transobturator slings were used in the studies that we analyzed for these variables.^55^ ^56^ ^57^ There was no significant difference between the groups for rates of overactive bladder, erosion, UTI, return to the operating room due to urinary retention, urinary retention for more or less than 6 weeks and de novo urgency.

The data on adverse events, leg pain, hematoma, return to the operating room due to erosion, blood loss, urethral perforation and vaginal perforation were all described in a single study, thus precluding a meta-analysis.

## Discussion

Several techniques have been described for the surgical treatment of SUI. Burch retropubic colposuspension surgery, considered the gold standard in the treatment of this condition for decades, gave way to the pubovaginal sling, and later, to the retropubic synthetic midurethral sling, described in 1996 by Ulmsten et al,^5^ showing a very satisfactory success rate. Next, transobturator slings were introduced using both outside-in and inside-out techniques as described by Delorme, in 2001,^10^ and de Leval, in 2003,^11^ respectively, in an attempt to reduce adverse events, especially bladder perforation and visceral and vascular lesions. In 2006, a single incision sling was developed following a trend toward minimally invasive procedures to reduce the amount of synthetic material used and reduce the blind needle path, thus minimizing tissue damage and infections.

Due to the large number of articles found in the literature, we decided to select studies of high-scientific quality to perform our meta-analysis.

In our work, we selected articles comparing midurethral sling versus Burch colposuspension, pubovaginal sling versus Burch colposuspension, pubovaginal sling versus midurethral sling, retropubic midurethral sling versus transobturator midurethral sling, outside-in transobturator midurethral sling versus inside-out transobturator midurethral sling, and minisling versus any other slings. The group with the highest number of articles was the one that compared the retropubic midurethral sling versus transobturator, with 22 studies selected.

Our view is that some bias should be considered while analyzing the results of this meta-analysis. Several studies do not distinguish between patients with and without intrinsic sphincteric deficiency, patients with recurrent or untreated SUI, which hinders a more detailed analysis. Another important bias was surgery performed concomitantly with surgical treatment for SUI (vaginal and abdominal hysterectomy, correction of anterior and posterior wall prolapses, and correction of vaginal vault prolapse). Moreover, we do not always find data on the individual conditions of the patients (lung disease, diabetes, neuropathy, etc.). The various criteria used for objective cure are also a bias factor. In the studies selected for our meta-analysis, the cure was defined based on urodynamic evaluation, a stress test and/or pad test. This lack of uniformity can significantly affect results. The same can be said regarding subjective cure, since some studies used quality of life questionnaires, while others only applied satisfaction surveys to define this outcome. Many of the studies analyzed are multicentric, with patients being operated on by different surgeons with varying experience degrees. It is known that surgeon experience is a determining factor for the success of a surgical procedure, as well as the occurrence of complications.^62^ ^63^

One of the factors that could be considered when choosing the treatment is the cost-benefit ratio. However, very few studies analyzed this variable. Among the studies selected for our meta-analysis, only one^19^ included such an evaluation.

As for the comparison of techniques in the analyzed studies, we verified that there was no significant difference regarding objective and subjective cure between midurethral sling and Burch colposuspension, although the latter presented more complications in terms of surgical wounds and UTI. With respect to midurethral slings, there were higher rates of bladder perforation and vaginal erosion, noting that comparisons were made with retropubic slings.

However, when comparing the pubovaginal sling and Burch surgery, the former was superior, in relation to objective and subjective cure, but presented a higher rate of return to the operating room due to urinary retention, which corroborates the literature results that pubovaginal slings are more retentionist.^64^ ^65^ We must point out that whenever the patient has indication for gynecological surgery using the abdominal route combined with stress urinary incontinence, Burch colposuspension is an adequate option.

In the comparison of pubovaginal sling versus midurethral sling, both presented high rates of objective cure but no significant difference between the two.

Comparing retropubic and transobturator slings, we observed that the retropubic devices were significantly superior, in relation to objective and subjective cure, despite the small difference. One possible explanation for this result is the more vertical positioning of the tape from the urethral axis in the retropubic route, unlike the horizontal position used via the transobturator route.^66^ This hypothesis would also explain the greater effectiveness of the retropubic technique over the transobturator in cases of SUI with IDS^67^ as well as the better long-term results favorable to retropubic sling.^29^ With regard to adverse events, we found a greater number of cases of bladder perforation, urinary retention, return to the operating room due to urinary retention, vascular injury and hematoma with retropubic slings. These last complications occur due to the blind passage of the needle through the Retzius space, which can lead to injury of veins and arteries, and ultimately bleeding and hematoma, as found in an ultrasound investigation immediately after surgery.^68^ The higher rate of urinary retention in retropubic slings is probably due to the more vertical position of the tape compared with the transobturator sling,^28^ ^69^ ^70^ as previously mentioned.

The transobturator sling, on the other hand, presented significantly more cases of leg pain, groin pain, neurological lesions and vaginal perforations.

Although the retropubic sling had significantly higher cure rates compared with the transobturator, the difference was small. The choice should therefore be based on the patient's history and individual characteristics, leaving the surgeon to decide the best route based on the possibility of complications, and his or her experience and preference, sharing the decision with the patient.

The TOT, when compared with TVT-O, did not show significant differences regarding objective and subjective cure. However, there was more vaginal perforation and erosion in the TOT group, which probably occurs because the needle passes closer to the vaginal sulcus in this technique.^40^

Compared with other slings, minislings did not show significant difference regarding objective cure; however, there was a significant difference regarding subjective cure, favorable to other slings. For adverse events, the group of other slings had a higher rate of groin pain and unspecified pain, which was only seen in transobturator slings.

In several comparisons, our meta-analysis failed to demonstrate significant differences regarding objective cure, subjective cure, and adverse effects among the various techniques, a result also obtained in a Cochrane meta-analysis published in 2015.^71^ Novara et al (2010),^72^ in turn, found superiority of retropubic slings compared with transobturator slings with respect to objective cure, and no difference between techniques related to subjective cure.

Our meta-analysis does not offer final conclusions about the effectiveness of the various techniques for intrinsic sphincteric deficiency, since most of the included studies failed to analyze this condition alone.

## Conclusion

Our systematic review, followed by the meta-analysis, included studies of high methodological quality aiming at comparing the various techniques available for surgical correction of SUI. According to our results, pubovaginal slings demonstrated better objective and subjective results when compared with Burch colposuspension surgery, but pubovaginal slings exhibited more retention, often resulting in a return to the operating room. When we compared the retropubic and transobturator slings, we observed the superiority of the retropubic sling objectively and subjectively but a greater number of adverse events. In the comparative analysis between minislings and other slings, superiority was noted for the latter in the subjective aspect. When comparing the midurethral slings with Burch colposuspension surgery, t no statistically significant difference in relation to objective or subjective cure was found. When comparing pubovaginal and midurethral slings, there was also no significant difference in relation to the objective cure. Likewise, no statistically significant difference was observed between inside-out and outside-in transobturator slings for both objective and subjective cure. Based on the above, we believe that the choice of technique should be aligned with several factors, such as abdominal or vaginal surgeries performed concomitantly, the surgeon's experience, the patient's prior surgeries, adverse events and availability of materials.
